# Homœopathy and Regular Medicine

**Published:** 1871-08

**Authors:** 


### Homœopathy and Regular Medicine.

Our correspondent upon this subject submits some remarkable inquiries and sratements. If any of our readers think that the questions require answer, or the statements correction, affirmation or denial, they will oblige by sending copy. For ourselves, we propose to submit it as offered, and let our readers do what they please with it.

